# Determining optimal clinical target volume margins in high-grade glioma based on microscopic tumor extension and magnetic resonance imaging

**DOI:** 10.1186/s13014-021-01819-0

**Published:** 2021-06-07

**Authors:** Shulun Nie, Yufang Zhu, Jia Yang, Tao Xin, Song Xue, Xianbin Zhang, Jujie Sun, Dianbin Mu, Yongsheng Gao, Zhaoqiu Chen, Xingchen Ding, Jinming Yu, Man Hu

**Affiliations:** 1 Department of Radiation Oncology, Shandong First Medical University and Shandong Academy of Medical Sciences, Qingdao Road 6699, Jinan, 250117 Shandong People’s Republic of China; 2 Department of Radiation Oncology, Shandong Cancer Hospital and Institute, Shandong First Medical University and Shandong Academy of Medical Sciences, Jiyan Road 440, Jinan, 250117 Shandong People’s Republic of China; 3 Department of Neurosurgery, Shandong Cancer Hospital and Institute, Shandong First Medical University and Shandong Academy of Medical Sciences, Jinan, Shandong People’s Republic of China; 4 Department of Neurosurgery, The First Affiliated Hospital of Shandong First Medical University, Jinan, Shandong People’s Republic of China; 5 Department of Pathology, Shandong Cancer Hospital and Institute, Shandong First Medical University and Shandong Academy of Medical Sciences, Jinan, Shandong People’s Republic of China; 6 Department of Radiology, Shandong Cancer Hospital and Institute, Shandong First Medical University and Shandong Academy of Medical Sciences, Jinan, Shandong People’s Republic of China

**Keywords:** High-grade glioma, Macropathology, Microscopic extension, Predictive model, Clinical target volume, Radiotherapy

## Abstract

**Introduction:**

In this study, we performed a consecutive macropathologic analysis to assess microscopic extension (ME) in high-grade glioma (HGG) to determine appropriate clinical target volume (CTV) margins for radiotherapy.

**Materials and methods:**

The study included HGG patients with tumors located in non-functional areas, and supratotal resection was performed. The ME distance from the edge of the tumor to the microscopic tumor cells surrounding brain tissue was measured. Associations between the extent of ME and clinicopathological characteristics were evaluated by multivariate linear regression (MVLR) analysis. An ME predictive model was developed based on the MVLR model.

**Results:**

Between June 2017 and July 2019, 652 pathologic slides obtained from 30 HGG patients were analyzed. The mean ME distance was 1.70 cm (range, 0.63 to 2.87 cm). The MVLR analysis identified that pathologic grade, subventricular zone (SVZ) contact and O^6^-methylguanine-DNA methyltransferase (MGMT) methylation, isocitrate dehydrogenase (IDH) mutation and 1p/19q co-deletion status were independent variables predicting ME (all *P* < 0.05). A multivariable prediction model was developed as follows: Y_ME_ = 0.672 + 0.513X_Grade_ + 0.380X_SVZ_ + 0.439X_MGMT_ + 0.320X_IDH_ + 0.333X_1p/19q_. The *R-square* value of goodness of fit was 0.780. The receiver operating characteristic curve proved that the area under the curve was 0.964 (*P* < 0.001).

**Conclusion:**

ME was heterogeneously distributed across different grades of gliomas according to the tumor location and molecular marker status, which indicated that CTV delineation should be individualized. The model could predict the ME of HGG, which may help clinicians determine the CTV for individual patients.

*Trial registration* The trial was registered with Chinese Clinical Trial Registry (ChiCTR2100046106). Registered 4 May 2021-Retrospectively registered.

**Supplementary Information:**

The online version contains supplementary material available at 10.1186/s13014-021-01819-0.

## Introduction

High-grade glioma (HGG) is the most commonly diagnosed primary brain tumor, and has a remarkable tendency to infiltrate the surrounding brain tissue. To protect brain function, gross total resection through surgery becomes almost impossible. Therefore, radiotherapy (RT) has become the main treatment for HGG patients. In National Comprehensive Cancer Network (NCCN) guidelines, for the delineation of a clinical target volume (CTV), a margin accounting for subdiagnostic tumor infiltration, of 1–2.5 cm for HGG in terms of the volumetric expansion of the gross target volume (GTV) is recommended. This is empirically determined, based on data demonstrating that over 80% of recurrences occur within a 2 cm margin of the contrast-enhanced lesion on computed tomography (CT) or magnetic resonance imaging (MRI) [1–4]. Thus far, this evidence is just indirect and inadequate [5]. Direct evidence for CTV delineation should be provided by the infiltration margin of the tumor. However, assessing the microscopic extension (ME) in HGG is challenging.

Pathology, as the gold standard of diagnosis, can precisely evaluate the ME of tumors. Unfortunately, it is difficult to obtain an adequate surgical margin for HGG, since the tumor is generally removed piecemeal under microscopy. Therefore, few previous studies have revealed the extent of the peripheral infiltration margin of glioma cells (GCs). Through macropathology, Mangiola et al. [6] found that GCs with a high migratory capability could widely distribute within the range of 1–2 cm from the edge of the tumor. This two-dimensional study is limited to one brain histological section per case and lacks detailed data for CTV delineation. Two histopathologic studies [7, 8] further showed that GCs could deeply infiltrate outside MRI abnormalities, which revealed that HGG had a potential tendency to invade further. However, this limited information does not provide precise evidence for target delineation.

With the development of genomics, O^6^-methylguanine-DNA-methyltransferase (MGMT) promotes methylation, isocitrate dehydrogenase (IDH) mutation and the co-deletion of chromosome arms 1p and 19q (1p/19q) have been proven to be strongly associated with the clinical behavior, response to therapy and outcome of HGG [9–11]. Unfortunately, to the best of our knowledge, the relationship between the ME and these molecular alterations has not yet been elucidated.

Therefore, the purpose of the present study was to identify the spatial ME of HGG according to consecutive macropathology, analyze its association with malignant factors including grade, tumor volume (V_tumor_), location, peritumoral brain edema (PTBE) and molecular markers, and create a model, that could provide evidence for more precisely determining the ME and, hence, the individual CTV to be applied in RT.

## Materials and methods

### Patient selection

This study involved HGG patients who underwent tumor resection at Shandong Cancer Hospital or the First Affiliated Hospital of Shandong First Medical University between June 2017 and July 2019. The inclusion criteria were as follows: (1) age ≥ 18 years; (2) preoperative Karnofsky Performance Status (KPS) ≥ 80; (3) unifocal enhancing tumor on T1-weighted MRI; (4) tumor located in nonfunctional area and successful supratotal resection (STR); and (5) tumor removal achieved with resection margins that included the neighboring normal tissue (between 2 and 3 cm away from the tumor border) (more detailed of the surgical methods of STR are provided in the Supplementary Methods). The exclusion criteria included: (1) a medical history of brain chemoradiotherapy and (2) multicentric or multifocal cerebral lesions. All tumors were graded according to the 2016 World Health Organization (WHO) classification [12]. This study was approved by the institutional review board of Shandong Cancer Hospital and Institute. All patients provided written informed consent to participate in the study.

### Preoperative MRI acquisition

Before any treatment, gadolinium-enhanced MRI examination was performed in all patients. MRI scanning was acquired using a 3 T MRI scanner (Philips Achieva 3 T). The scanning protocols were obtained with the following parameters. T1-weighted imaging: echo time (TE) = 10 ms, repetition time (TR) = 495 ms, slice thickness/gap = 3 mm/0 mm, number of signal averaged (NSA) = 1, field of view (FOV) = 260 mm × 260 mm, matrix = 256 × 256. T2-weighted imaging: TE = 110 ms, TR = 13312 ms, slice thickness/gap = 3 mm/0 mm, NSA = 1, FOV = 260 mm × 260 mm, matrix = 416 × 416. T2-fluid attenuated inversion recovery (T2-FLAIR): TE = 120 ms, TR = 11000 ms, slice thickness/gap = 3 mm/0 mm, NSA = 1, FOV = 260 mm × 260 mm, matrix = 320 × 320. To accurately match the MRI and the tissue specimen, the orbitomeatal line (OML) was perpendicular to the scanning table.

**Fig. 1:**
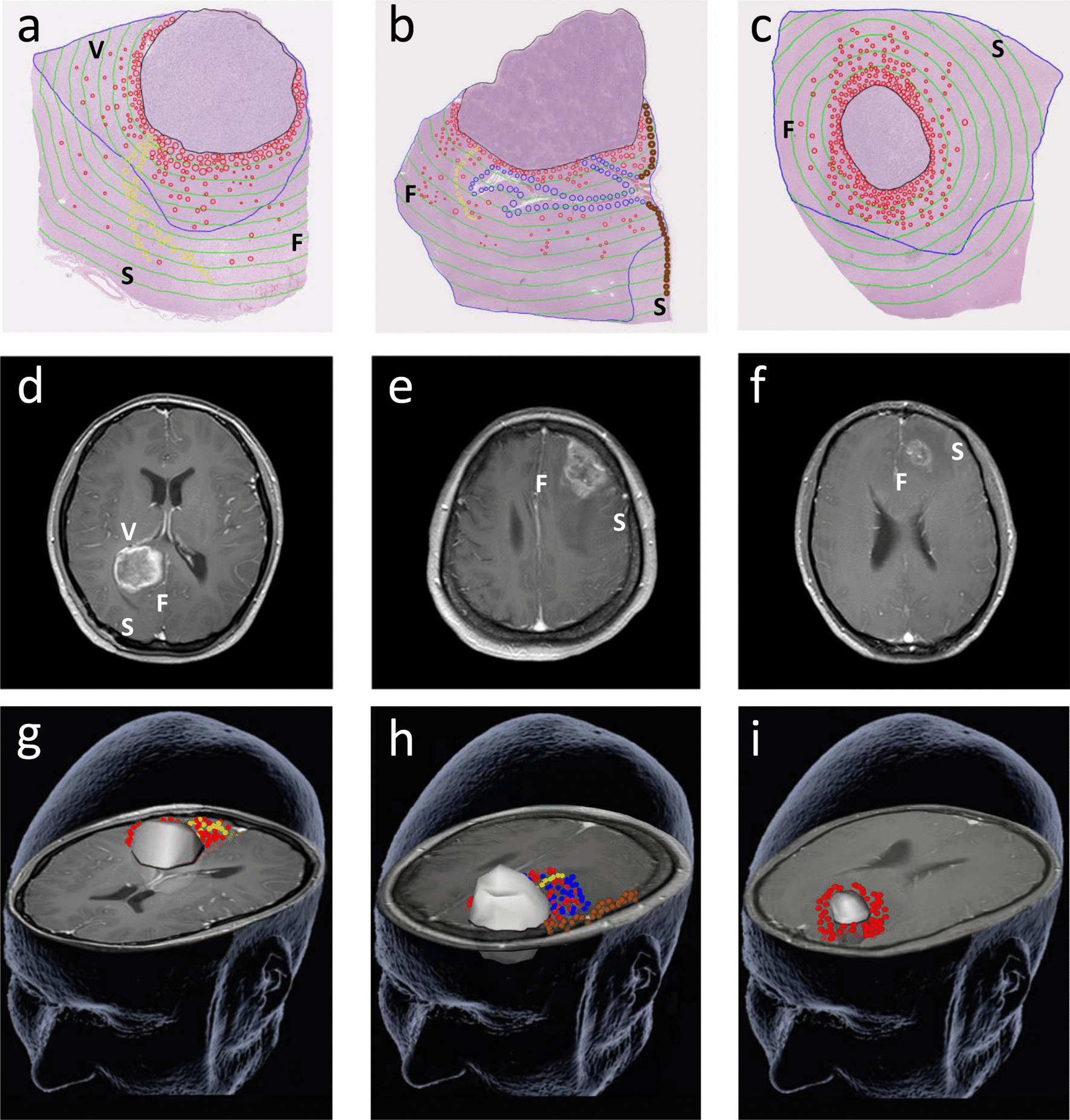
Tumor microscopic extension distribution for different locations based on preoperative T1-weighted MRI. **a**, **d**, **g** Type I; **b**, **e**, **h** Type II; **c**, **f**, **i** Type III. At the sites where the glioma cells produced direct invasion, perineural spread, subpial growth and perivascular spread are outlined in red, yellow, brown and blue, respectively. Black line: tumor-containing area; blue line: PTBE-containing area; green line: 0.25 cm intervals. *S* skull; *V* ventricle; *F* falx

Preoperative MRI was evaluated by two senior radiologists and the data (T2-FLAIR and T1-weighted sequence) were used to determine the PTBE volume (V_PTBE_), V_tumor_ and tumor localization. The V_tumor_ was defined as the area of increased signal intensity on contrast-enhancing T1-weighted sequence. The V_PTBE_ was defined as the area of FLAIR hyperintensity signal seen on T2-weighted sequence beyond the contrast-enhanced T1-weighted images. Based on the spatial relationship between the tumor and the subventricular zone (SVZ) and cortex, the tumor location was classified as follows: Type I, tumor contacting SVZ; Type II, tumor involving cortex; Type III, tumor neither contacting SVZ nor infiltrating cortex (Fig. 1).

### Surgical specimen processing

**Fig. 2:**
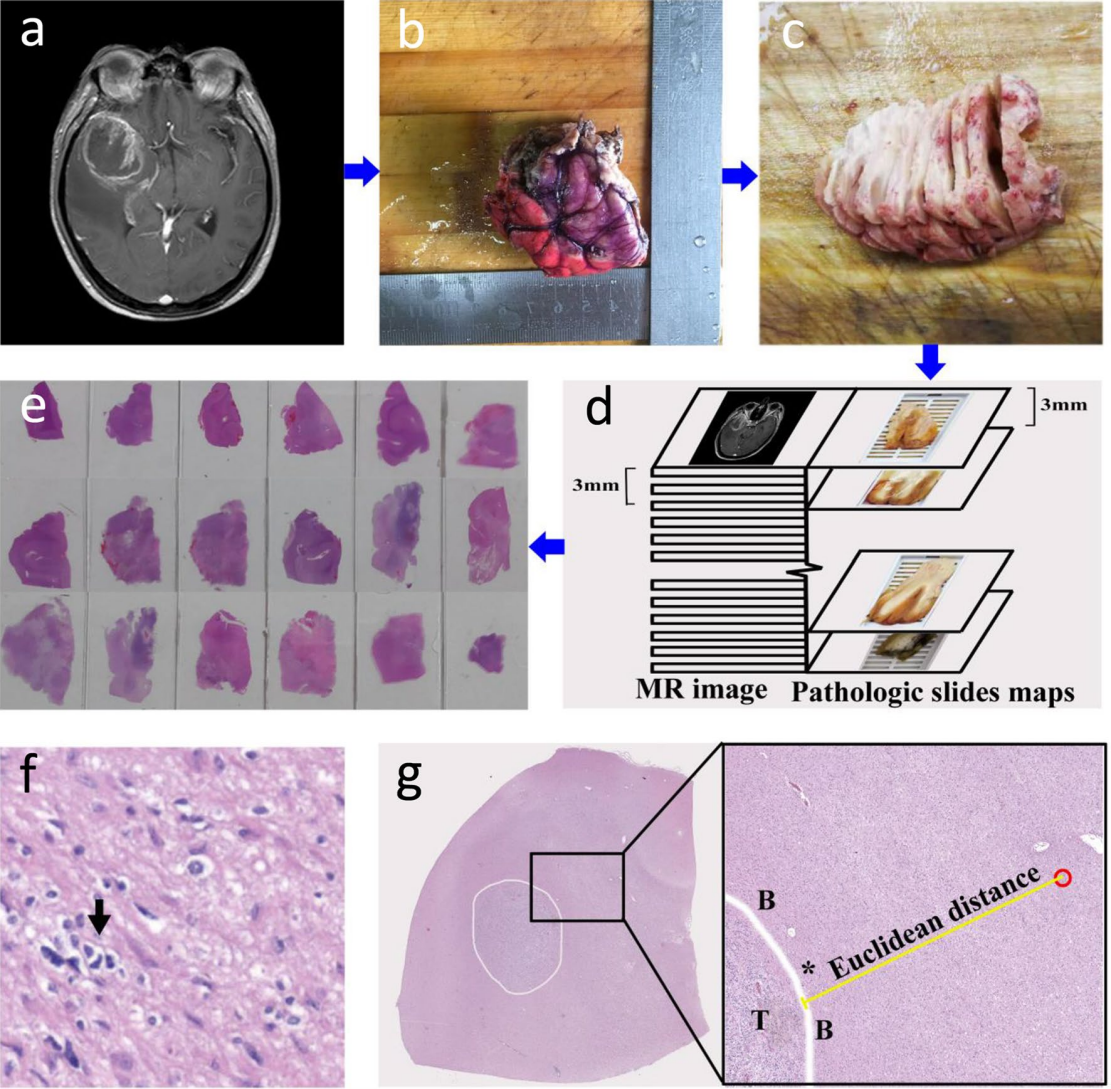
**a**–**e** Pathology procedure. First, by referring to MRI (**a**), the surgical specimen was oriented according to the in vivo geometry, and the colored edges indicate the direction of the specimen in the brain (**b**). Afterward, the fixed specimen was sectioned consecutively at approximately 3 mm intervals (**c**), which ensured that each specimen slice matched the MRI slice (**d**). Finally, the tissue specimen was selected for H&E (**e**). **f** Histologic features of areas surrounding the glioma: An atypical cell with a moderately irregular nucleus suspected of neoplasia (arrow) is shown. **g** H&E staining cross-Sect. (5 × magnification) shows a wide view of the specimen tissue section with the tumor outlined in white. Magnified H&E view (10 × magnification) shows invasive glioma cells (outlined in red) outside the boundary (B) of the tumor (T). The ME (Euclidean) distance from the edge of the tumor (*) to the microscopic tumor cells surrounding brain tissue was measured (yellow line)

After resection, the plane of the OML was marked on the specimen. Subsequently, the surgical specimen was oriented according to the in vivo geometry, marked with different colors to indicate the original orientation of the specimen in the brain and fixed in 10% formalin (≥ 24 h). The dimensions of the tumor samples, both before and after fixation were documented to determine the reduction in size due to fixation (Additional file 1: Table S1). Then, the plane of the OML was perpendicular to the table; the specimen was cut using a tissue slicer from the cranial to the caudal side in approximately 3 mm thick slices, which ensured that each specimen slice could match the MRI slice. The slices were contiguous, and their individual thickness was measured with a ruler. Finally, whole-mount paraffin sections were made and cut into 5 µm sections per slice, which were stained with hematoxylin and eosin (H&E) (Fig. 2a–e). In addition, each patient underwent molecular testing, and the methods used for analyzing the methylation status of the MGMT promoter and determining the mutational status of IDH by DNA pyrosequencing have been described previously [13, 14]. Deletions of chromosomes 1p/19q were evaluated by fluorescence in situ hybridization analysis in tumor tissue sections [13, 14].

### ME analysis and measurement

The tumor-containing area and the PTBE area in the H&E sections were microscopically outlined, scanned and recorded by TissueFAXS PLUS (TissueGnostics, Austria). Subsequently, the scan images were imported into Photoshop (Adobe Systems, USA) to identify the microscopic evidence by two experienced pathologists who were blinded to the clinical data. Invasive GCs were identified by means of their nuclear atypia and heteropyknotic staining [6] (Fig. 2f). To measure the spatial distribution of invasive GCs, pathologic slices were used to generate three-dimensional (3D) graphics through 3D-DOCTOR (Able Software Corp, USA). First, the contours of individual H&E sections were digitized and recorded to generate a 3D surface of the reconstructed specimen. Second, the 3D specimens were correct for retraction through scaling parameters (Additional file 1: Table S1). Then, the corrected 3D image was registered to preoperative T1-weighted MRI using the outline of tumor to perform point-based registration. After the above steps, the ME distance and direction of the GCs relative to the primary tumor bulk were established. In the in-slice direction, the nearest (Euclidean) distance [15] from the edge of the tumor to the microscopic GCs surrounding brain tissue was measured by Photoshop (Fig. 2g). In the through-slice direction, the number of slices from the invasive GCs to the lesion border was counted and multiplied by the slice thickness (× 3 mm). The ME of each slice is defined as the maximal distance of the ME. The ME of each patient was defined as the maximum ME across different slices (Additional file 1: Table S2).

### Statistical analysis

For all analyses, we used SPSS 22.0 (IBM Armonk, NY, USA), and values for which *P* < 0.05 were considered statistically significant. Categorical variables were expressed as proportions. Continuous variables were expressed as mean ± standard deviation or median (interquartile range), as appropriate. The difference between two groups was assessed with *Student’s* t-test or *Chi-Squared* test. When comparing more than two variables, we performed one-way analysis of variance. Post-hoc analysis was used to compare pairwise differences. Spearman’s rank correlation was performed to evaluate the relationship of the ME with the grade, V_tumor_, location, V_PTBE_ and molecular marker status. A multivariate linear regression (MVLR) model was created from variables with a *P* < 0.05 on correlation analysis, using stepwise regression. To assess the prediction efficiency of this model, calibration was evaluated using the *R*-square *goodness-of-fit* test, and discrimination was evaluated using receiver operating characteristic (ROC) curves with the corresponding area under the curve (AUC).

## Results

### Patient and tumor characteristics

Thirty patients were included of 32 recruited (2 withdrew after consent). In total, 652 H&E slides were analyzed in this study. The characteristics of the patients are listed in Table 1. The details about baseline, immediate post-operative and long-term neurological symptoms of patients are shown in Additional file 1: Table S3. Histological analysis revealed that 17 patients had grade III gliomas and 13 patients had grade IV gliomas. The tumor specimen and its radiologic images were almost identical in their morphology. Further analysis revealed that the volume of HGG was similar on T1-weighted MRI and in specimens (24.03 ± 20.54cm^3^ vs. 27.14 ± 22.80cm^3^, *P* = 0.581).

**Table 1:** Baseline characteristics of HGG patients

Characteristic	No. (%)
Gender	
Male	16 (53.3)
Female	14 (46.7)
Age (years)	
≤ 60	24 (80)
> 60	6 (20)
Preoperative KPS	
80–90	8 (26.7)
≥ 90	22 (73.3)
Pathologic grade	
Grade III	17 (56.7)
Grade IV	13 (43.3)
Tumor volume (cm^3^)	
Median (IQR)	20.04 (10.68–42.92)
Range	1.83–80.66
PTBE volume (cm_3_)	
Median (IQR)	105.26 (56.92–137.37)
Range	17.2–235.65
Tumor location	
Type I	11 (36.7)
Type II	9 (30)
Type III	10 (33.3)
Molecular markers	
MGMT methylation status	
Unmethylated	12 (40)
Methylated	18 (60)
IDH mutation status	
Mutated	12 (40)
Wild type	18 (60)
1p/19q co-deletion status	
Co-deleted	11 (36.7)
Non-co-deleted	19 (63.3)

*HGG* high-grade glioma; *KPS* Karnofsky performance status; *IQR* interquartile range; *PTBE* peritumoral brain edema; *MGMT* O^6^-methylguanine-DNA-methyltransferase; *IDH* isocitrate dehydrogenase; *1p/19q co-deletion* the co-deletion of chromosome arms 1p and 19q

### Pathologic ME characteristics

**Fig. 3:**
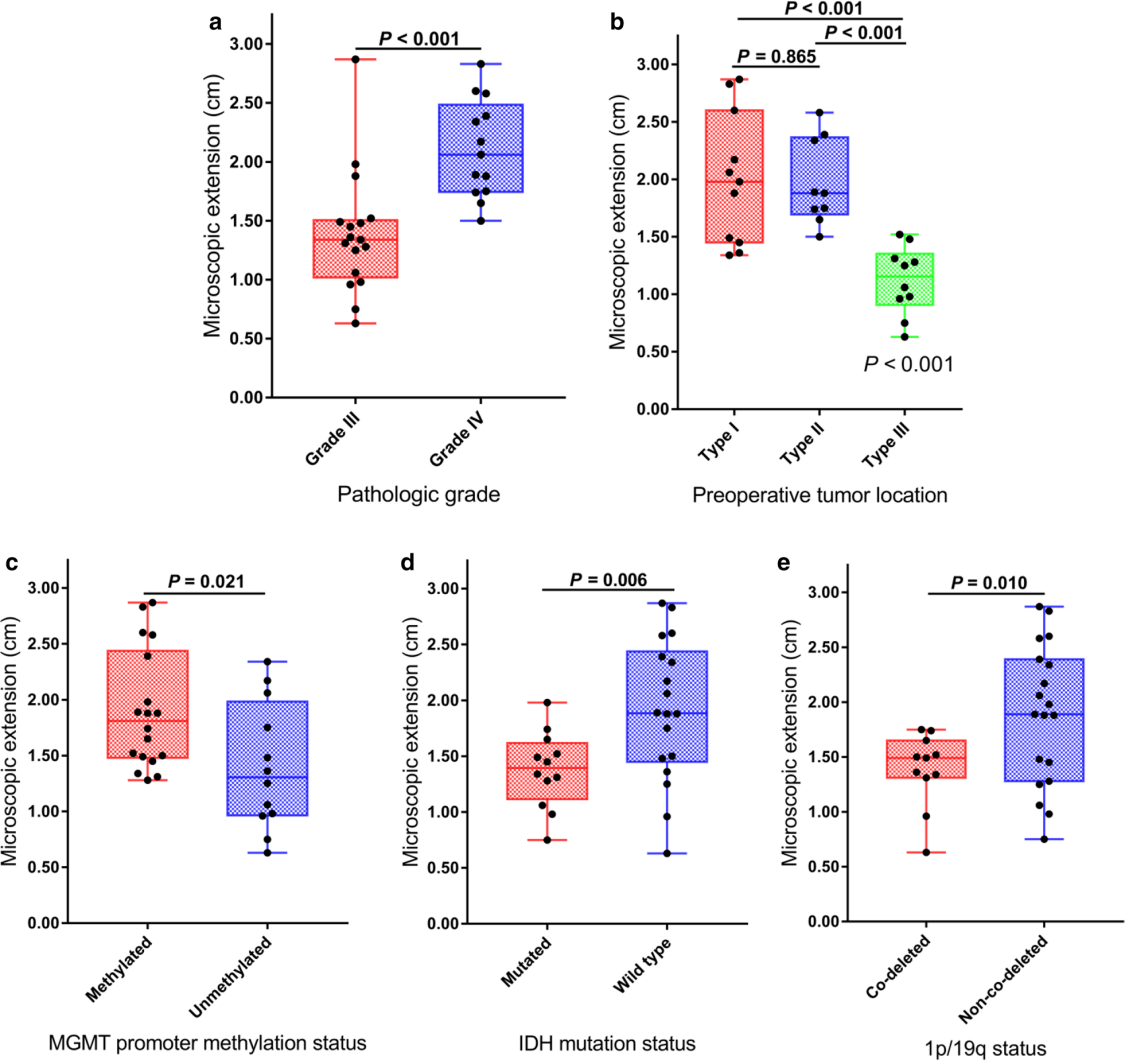
Box plot analysis showing the microscopic extension in different subgroups. **a** Pathologic grade; **b** preoperative tumor location; **c** MGMT promoter methylation status; **d** IDH mutation status; **e** 1p/19q co-deletion status

We demonstrated obvious differences in the ME among individuals (Additional file 1: Table S2). The GCs were heterogeneously distributed through direct invasion, skip metastases, or along neural fiber tracts, pia mater and basement membranes of blood vessels. The mean ME (ME_mean_) distance was 1.70 cm (range, 0.63 to 2.87 cm). Grade IV gliomas had significantly higher ME than grade III tumors (2.11 ± 0.42 cm vs. 1.39 ± 0.52 cm, *P* < 0.001) (Fig. 3a). A significant correlation was found between the extent of ME and pathologic grade (*P* < 0.001). However, there was no statistically significant correlation between the extent of ME and V_tumor_ (*P* = 0.779) (Table 2).

**Table 2:** Correlation between ME extent and the different variables

Variables	ME extent	
	*r* value*	*P* value*
Pathologic grade		
III/IV	0.668	< 0.001
Tumor volume	0.053	0.779
SVZ contact		
No/Yes	0.384	0.036
Cortical involvement		
No/Yes	0.370	0.044
PTBE volume	0.061	0.751
MGMT methylation status		
Unmethylated/Methylated	0.417	0.022
IDH mutation status		
Mutated/Wild type	0.448	0.013
1p/19q co-deletion status		
Co-deleted/Non-co-deleted	0.372	0.043

^*^r and* P* value according to the spearman’s rank correlation

*ME* microscopic extension; *SVZ* subventricular zone; *PTBE* peritumoral brain edema; *MGMT* O^6^-methylguanine-DNA-methyltransferase; *IDH* isocitrate dehydrogenase; *1p/19q co-deletion* the co-deletion of chromosome arms 1p and 19q

### Relationship between extent of ME and tumor location

The typical ME distributions in the different locations are shown in Fig. 1. Invasive GCs from type I were widely distributed in normal brain tissue, the ME distance was 2.00 ± 0.57 cm, and in type II, subpial growth became a main pathway for GCs to distant invasion, and the ME distance was 1.97 ± 0.37 cm; whereas in type III, infiltration occurred in the border of the primary lesion with an ME value of 1.12 ± 0.30 cm. The ME of type I or type II was significantly higher than that of type III (both *P* < 0.001). However, the ME difference between type I and type II did not reach statistical significance (*P* = 0.865) (Fig. 3b). Furthermore, a significant positive correlation was found between the extent of ME and SVZ contact or cortical involvement (*P* = 0.036 and 0.044, respectively) (Table 2).

### Relationship between extent of ME and PTBE

PTBE infiltration was found in all patients. Meanwhile, we observed that GCs invaded beyond the PTBE area in 40% (12/30) of patients, including 7 with perineural spread and 5 with subpial growth (Fig. 1a, b). In contrast, the invasive GCs from 60% (18/30) of patients were only contiguous with the lesion (Fig. 1c) and showed a much smaller ME range than the PTBE area (24.98 ± 14.80cm^3^ vs. 100.75 ± 52.48cm^3^, *P* = 0.017). Spearman’s rank correlation analysis revealed no significant relationship between the extent of ME and V_PTBE_ (*P* = 0.751) (Table 2).

### MGMT, IDH and 1p/19q status impact the ME of glioma

As shown in Fig. 3c–e, MGMT methylated tumors had a significantly higher ME than their unmethylated counterparts (1.90 ± 0.53 cm vs. 1.40 ± 0.57 cm, *P* = 0.021). In contrast, IDH mutated tumors had a lower ME than IDH wild-type tumors (1.38 ± 0.34 cm vs. 1.91 ± 0.64 cm, *P* = 0.006). Tumors with 1p/19q co-deletion had a lower ME than those with 1p/19q non-co-deletion (1.39 ± 0.34 cm vs. 1.88 ± 0.64 cm, *P* = 0.010). A significant correlation was found between the extent of ME and MGMT, IDH and 1p/19q status (*P* = 0.022, 0.013 and 0.043, respectively) (Table 2).

### Predictive model analysis 

**Fig. 4:**
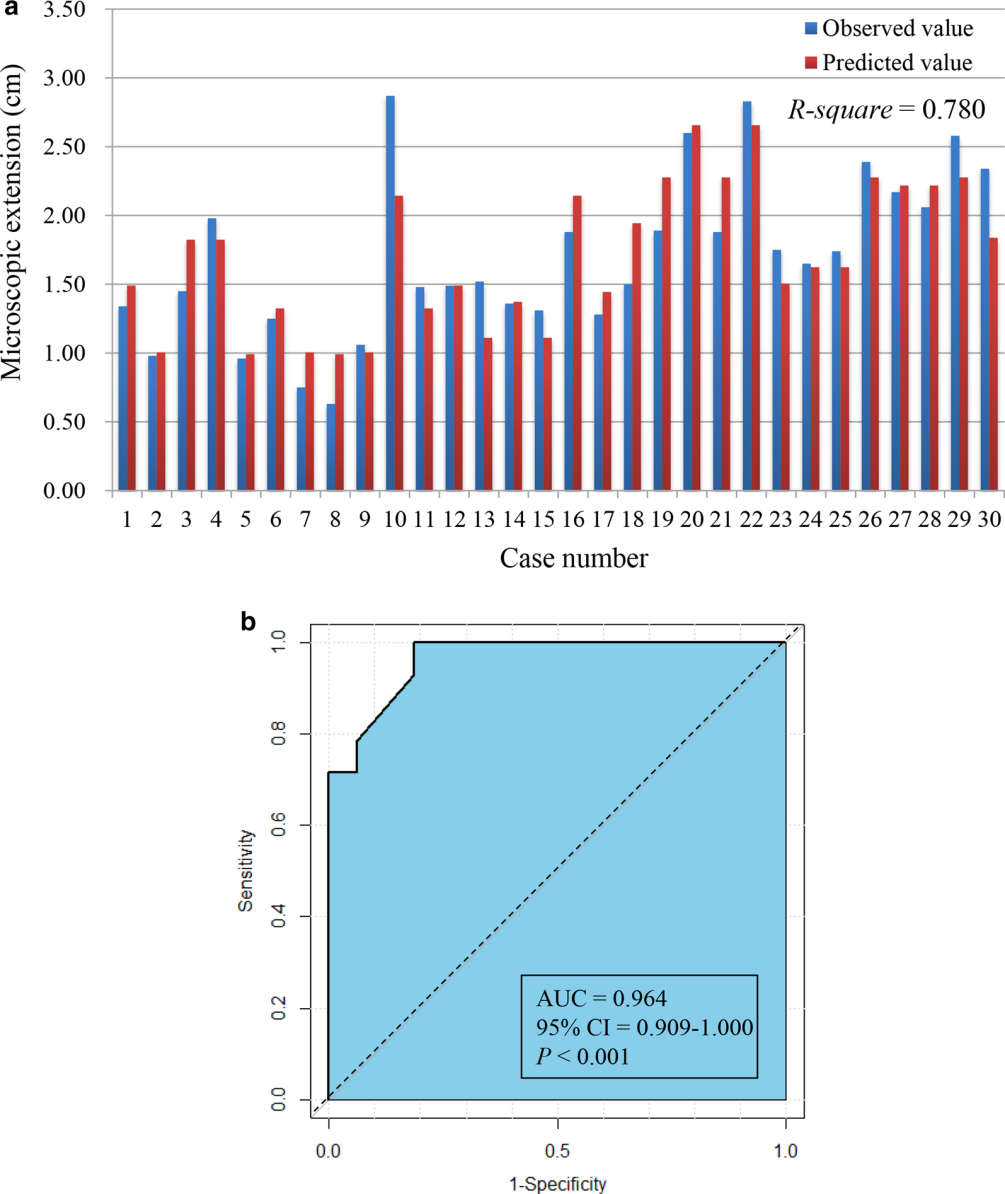
**a** Histogram plot showing the calibration of the predictive model; **b** ROC curves depicting the predictive discrimination of the model

The MVLR analysis identified that grade, SVZ contact and MGMT, IDH and 1p19q status were independent variables predicting ME (all *P* < 0.05), with grade having the largest β-coefficient (0.513) (Table 3). A predictive model was created as follows: Y_ME_ = 0.672 + 0.513X_Grade_ + 0.380X_SVZ_ + 0.439X_MGMT_ + 0.320X_IDH_ + 0.333X_1p/19q_. The model was evaluated with good performance in terms of calibration, with the *R-square* value of the goodness-of-fit test being 0.780 (Fig. 4a). Meanwhile, we used the ME_mean_ value (1.70) as a cutoff to evaluate the discrimination of the model, which proved that the AUC was 0.964 (95% confidence interval [CI]: 0.909–1.000, *P* < 0.001) (Fig. 4b).

**Table 3:** Variables associated with ME extent in multivariate linear regression model

Variable	β	SE	95% CI for β	*P* value	Score (0, 1)
Pathologic grade	0.513	0.136	0.232–0.794	0.001	
Grade III					0
Grade IV					1
SVZ contact	0.380	0.124	0.125–0.635	0.005	
No					0
Yes					1
Molecular markers					
MGMT methylation status	0.439	0.129	0.171–0.706	0.002	
Unmethylated					0
Methylated					1
IDH mutation status	0.320	0.141	0.028–0.611	0.033	
Mutated					0
Wild type					1
1p/19q co-deletion status	0.333	0.120	0.084–0.581	0.011	
Co-deleted					0
Non-co-deleted					1

*ME* microscopic extension; *β* regression coefficient; *SE* standard error; *CI* confidence interval; *SVZ* subventricular zone; *MGMT* O^6^-methylguanine-DNA-methyltransferase; *IDH* isocitrate dehydrogenase; *1p/19q co-deletion* the co-deletion of chromosome arms 1p and 19q

## Discussion

To our knowledge, this study is the first to determine the CTV margins of HGG based on consecutive macropathology. Our results showed that GC invasion into the surrounding brain tissue is complex and highly heterogeneous across different types of HGGs, according to grade, location and molecular markers. We built an easy-to-use model to guide individualized target delineation.

To date, there is a lack of radiologic–histopathologic correlation studies upon which a consensus can be made to guide the targeted delineation of HGG. Although MRI is proposed as the first choice for pretherapeutic and post-therapeutic evaluation of HGG due to its economic cost-effectiveness and high accuracy, its ability to determine the target volume is inconclusive. Our study found that the contrast-enhanced area on T1-weighted MRI reflects only the high-density region of GCs in macropathology, that is, the areas of blood–brain barrier disruption, as described in a previous study [16]. These areas are sufficient for defining the GTV of HGG. However, for CTV delineation, the ability of MRI is limited. Macropathology has its inherent advantages in determining the CTV, which can well make up for the deficiency of MRI. In our study, we redefined the CTV margins in HGG patients based on macropathology and MRI. We recommended that GTV was defined by T1-weighted abnormality on the MRI, which consisted of all postoperative-enhanced MRI and the surgical cavity. The CTV was defined as the GTV plus a margin, which was determined by our model, adjusted to anatomical borders. The CTV was expanded by 3 mm to create the respective planning target volume.

We identified and incorporated 5 independent clinical factors into the MVLR model, including grade, SVZ contact and MGMT, IDH and 1p/19q status. In our model, grade contributed the most to predicting ME of HGG. In agreement with the literature [14, 17, 18], the WHO grade system is consistently identified as an important factor for ME. We found that higher grade glioma was associated with greater ME. Another significant factor influencing the ME on multivariate analysis was preoperative tumor location. It is noteworthy that the invasive GC distribution was wider in tumors contacting the SVZ, which was consistent with the results of previous retrospective studies. Lim et al. [19] demonstrated that SVZ contact was significantly associated with multifocality. In a study by Adeberg et al. [20], glioblastoma that contacted the SVZ showed higher rates of distant progression and multifocal recurrence than noncontacting lesions. This finding may be explained by the recruitment of glioma stem-like cells in the SVZ, resulting in an aggressive glioma subtype [19–21]. In contrast, invasive GCs from type III were only contiguous with the lesion. In regard to the study of Adeberg et al. [20], a similar result was found glioblastoma recurrence always occurred in the border of the primary lesion in the tumor, which neither contacted the SVZ nor infiltrated the cortex. Based on these results, the determination of the CTV margin according to different locations has been proposed for the first time, but more evidence is still needed to inform clinical practice.

Further analysis revealed a relationship between the extent of ME and MGMT, IDH and 1p/19q status. We found that MGMT methylation induced invasion in distant locations compared with unmethylated cells. Our results confirmed two previous imaging studies [22, 23] and showed that methylated glioblastoma patients with MGMT had a greater tendency to develop out-of-field recurrence than those with unmethylated status. Interestingly, the present study also observed that IDH wild-type or 1p/19q non-co-deleted patients showed increased tumor migration and invasion compared with their counterparts, which has not been previously reported. These findings might help oncologists provide more tailored RT fields to patients with HGG.

It is highly controversial whether PTBE needs to be intentionally included in the CTV in glioma, since the relationship between the distribution of GCs and PTBE has still been undefined. In the present study, we first comprehensively revealed both relationships through macropathology and found that ME was not significantly associated with PTBE. We observed that the ME range of 60% of patients was much smaller than that of the PTBE area, whereas infiltration outside of the PTBE occurred when GCs spread along the perineural direction or subpial growth. Similar results were confirmed by Kelly et al. [24] through stereotactic biopsy and Yamahara et al. [7] through autopsy. Based on these important findings, we suggested that RT including the entire PTBE was not necessary. PTBE might merely coexist with infiltrating GCs in what is a spatial coincidence but actually reflects two independent processes; unreasonable RT fields would increase normal tissue toxicity, thereby influencing the prognosis of patients [4, 25].

PET has been shown to be useful in the detecting of diffuse glioma infiltration [26–29]. Through comparing histopathology and multimodal imaging, Verburg et al. [26] found that O-(2-[18F]-fluoroethyl)-L-tyrosine PET showed strong performance in detecting the infiltration of enhanced glioma. In another study, Kinoshita et al. [27] also found that ^18^F-FDG–^11^C-Methionine PET shows a better indicator for glioma cell infiltration. Considering the advantage of PET in reflecting the anisotropy of glioma extent [26], PET may be used as a non-invasively image integrated with our model to help the individualize target volume delineation in three dimensions. In our next study, we will further explore the feasibility of PET-based target volume delineation by combining the macropathology with PET imaging.

Our study has several limitations. First, the size of our study population was small; thus, a large cohort is needed to further develop our model. Second, this margin formula is not validated and should not be used clinically until further work is conducted and published. Third, our study analyzed only patients with good performance. An inherent bias is that tumors that are amenable to STR likely have anatomic, clinical, or biological characteristics that differ from the majority of tumors where subtotal resection is performed. Fourth, the CTV margins determined in this study is only isotropic margin. It is important to acknowledge that the anisotropy of disease extent in patients' brains is not accounted for with a single margin value. In our future study, we will develop a second formula that might help with the design of anisotropic margins.

In conclusion, tumor cells were heterogeneously distributed in different gliomas. Pathologic grade, location, MGMT, IDH and 1p/19q status were demonstrated to be important factors contributing to ME. This suggested that the delineation of CTV should be individualized. Using these factors, we first built an invasive risk score model, which can better provide valuable evidence to predict the ME of glioma, and this may help clinicians determine the CTV of patients.

## Supplementary Information

**Additional file 1: Table S1**. (a) Tumor size before and after fixation, corresponding area retraction ratio for each case; (b) Normal brain tissue size before and after fixation, corresponding area retraction ratio for each case. **Table S2**. Microscopic extension (ME) and prevalence of infiltrating cells for each patient. **Table S3**. Baseline, immediate post-operative and long-term neurological symptoms.

## Data Availability

The data used in this analysis is from publications available in the public domain.

## Abbreviations

HGG: High-grade glioma
NCCN: National comprehensive cancer network
CTV: Clinical target volume
GTV: Gross target volume
CT: Computed tomography
MRI: Magnetic resonance imaging
ME: Microscopic extension
MGMT: O^6^-methylguanine-DNA-methyltransferase
IDH: Isocitrate dehydrogenase
1p/19q: Chromosome arms 1p and 19q
PTBE: Peritumoral brain edema
KPS: Karnofsky performance status
STR: Supratotal resection
WHO: World Health Organization
TE: Echo time
TR: Repetition time
NSA: Number of signal averaged
FOV: Field of view
FLAIR: Fluid attenuated inversion recovery
OML: Orbitomeatal line
SVZ: Subventricular zone
MVLR: Multivariate linear regression
ROC: Receiver operating characteristic
AUC: Area under the curve
CI: Confidence interval
