# Supplementary material for: Determining optimal clinical target volume margins in high-grade glioma based on microscopic tumor extension and magnetic resonance imaging
Source: Radiat Oncol. 2021 Jun 7;16:97. doi: 10.1186/s13014-021-01819-0 (PMC8186169; doi:10.1186/s13014-021-01819-0)
Supplement: Supplementary file 1 — Additional file 1: Table S1. (a) Tumor size before and after fixation, corresponding area retraction ratio for each case; (b) Normal brain tissue size before and after fixation, corresponding area retraction ratio for each case. Table S2. Microscopic extension (ME) and prevalence of infiltrating cells for each patient. Table S3. Baseline, immediate post-operative and long-term neurological symptoms. [file 13014_2021_1819_MOESM1_ESM.docx]

**Supplementary Methods**

Surgery was performed in all patients with the aid of imaging and brain mapping techniques. Mapping of eloquent language and motor cortex was performed to define the cortical safe-entry zone. Tumor borders were defined by intraoperative microscope coupled with intraoperative MRI. After complete microsurgical resection of the enhancing tumor component, intraoperative neuronavigation or/and intraoperative neuromonitoring were done to ensure safe and successful supratotal resections (the resection margin was 2-3cm away from the tumor border). In all cases, the resection followed the principle of localizing the functional motor, language or visual tracts, which represents the limit of resection. After surgery, all patients underwent MRI examination (within 72 hours after surgery) to further confirm that each patient had achieved supratotal resections.

**Supplementary Table S1**

**(a)** Tumor size before and after fixation, corresponding area retraction ratio for each case

| Case no. | D_long_ | D_short_ | d_long_ | d_short_ | Area retraction |
| --- | --- | --- | --- | --- | --- |
| 1 | 5.63 | 4.92 | 5.09 | 4.25 | 0.78 |
| 2 | 1.80 | 1.52 | 1.56 | 1.29 | 0.74 |
| 3 | 2.49 | 2.15 | 2.06 | 1.83 | 0.70 |
| 4 | 3.89 | 2.99 | 3.89 | 2.99 | 1.00 |
| 5 | 4.14 | 3.21 | 4.14 | 3.21 | 1.00 |
| 6 | 4.42 | 4.19 | 3.98 | 3.63 | 0.78 |
| 7 | 4.08 | 2.73 | 3.94 | 2.67 | 0.94 |
| 8 | 4.81 | 3.90 | 4.67 | 3.58 | 0.89 |
| 9 | 4.88 | 4.02 | 4.88 | 4.02 | 1.00 |
| 10 | 4.97 | 4.82 | 4.68 | 4.51 | 0.88 |
| 11 | 2.90 | 1.61 | 2.77 | 1.43 | 0.85 |
| 12 | 6.58 | 6.14 | 5.83 | 5.46 | 0.79 |
| 13 | 4.33 | 3.03 | 4.33 | 3.03 | 1.00 |
| 14 | 1.80 | 1.67 | 1.80 | 1.67 | 1.00 |
| 15 | 4.41 | 4.30 | 4.09 | 3.86 | 0.83 |
| 16 | 6.74 | 4.35 | 6.28 | 4.01 | 0.86 |
| 17 | 3.14 | 2.83 | 3.14 | 2.83 | 1.00 |
| 18 | 1.73 | 1.51 | 1.68 | 1.38 | 0.89 |
| 19 | 2.15 | 1.78 | 1.85 | 1.34 | 0.65 |
| 20 | 3.69 | 2.92 | 3.02 | 2.49 | 0.70 |
| 21 | 2.33 | 1.80 | 2.24 | 1.68 | 0.90 |
| 22 | 4.94 | 4.18 | 4.94 | 4.18 | 1.00 |
| 23 | 5.69 | 3.59 | 5.55 | 3.47 | 0.94 |
| 24 | 3.14 | 2.75 | 2.44 | 2.16 | 0.61 |
| 25 | 4.19 | 3.14 | 3.57 | 2.78 | 0.75 |
| 26 | 2.77 | 2.48 | 2.77 | 2.48 | 1.00 |
| 27 | 4.60 | 4.51 | 4.13 | 4.03 | 0.80 |
| 28 | 4.83 | 3.45 | 4.83 | 3.45 | 1.00 |
| 29 | 4.67 | 3.15 | 4.67 | 3.15 | 1.00 |
| 30 | 3.27 | 3.06 | 2.88 | 2.47 | 0.71 |

**(b)** Normal brain tissue size before and after fixation, corresponding area retraction ratio for each case

| Case no. | D_long_ | D_short_ | d_long_ | d_short_ | Area retraction |
| --- | --- | --- | --- | --- | --- |
| 1 | 2.19 | 2.01 | 2.08 | 1.95 | 0.92 |
| 2 | 2.48 | 2.37 | 2.33 | 2.21 | 0.88 |
| 3 | 2.75 | 2.62 | 2.75 | 2.59 | 0.99 |
| 4 | 2.74 | 2.67 | 2.74 | 2.67 | 1.00 |
| 5 | 2.72 | 2.64 | 2.72 | 2.64 | 1.00 |
| 6 | 2.46 | 2.40 | 2.44 | 2.35 | 0.97 |
| 7 | 2.72 | 2.72 | 2.68 | 2.65 | 0.96 |
| 8 | 2.49 | 2.42 | 2.45 | 2.40 | 0.98 |
| 9 | 2.74 | 2.72 | 2.74 | 7.72 | 1.00 |
| 10 | 3.02 | 2.88 | 3.00 | 2.85 | 0.98 |
| 11 | 2.50 | 2.46 | 2.45 | 2.37 | 0.94 |
| 12 | 2.66 | 2.51 | 2.53 | 2.44 | 0.92 |
| 13 | 2.47 | 2.42 | 2.47 | 2.42 | 1.00 |
| 14 | 3.05 | 2.70 | 3.05 | 2.70 | 1.00 |
| 15 | 2.75 | 2.75 | 2.64 | 2.60 | 0.91 |
| 16 | 2.90 | 2.83 | 2.81 | 2.72 | 0.93 |
| 17 | 2.94 | 2.86 | 2.94 | 2.86 | 1.00 |
| 18 | 2.69 | 2.66 | 2.69 | 2.65 | 0.99 |
| 19 | 2.93 | 2.87 | 2.73 | 2.54 | 0.82 |
| 20 | 3.00 | 2.77 | 2.89 | 2.57 | 0.89 |
| 21 | 2.43 | 2.21 | 2.27 | 2.04 | 0.86 |
| 22 | 2.99 | 2.89 | 2.99 | 2.89 | 1.00 |
| 23 | 2.75 | 2.59 | 2.71 | 2.56 | 0.97 |
| 24 | 2.23 | 2.20 | 1.98 | 1.92 | 0.77 |
| 25 | 2.44 | 2.39 | 2.25 | 2.13 | 0.82 |
| 26 | 3.11 | 2.68 | 3.11 | 2.68 | 1.00 |
| 27 | 2.74 | 2.61 | 2.69 | 2.54 | 0.96 |
| 28 | 3.05 | 3.00 | 3.05 | 3.00 | 1.00 |
| 29 | 2.68 | 2.57 | 2.68 | 2.57 | 1.00 |
| 30 | 2.98 | 2.90 | 2.85 | 2.71 | 0.89 |

***Abbreviations*:** D_long_ = long diameter on transverse plane before formalin fixation; D_short_ = short diameter on transverse plane before formalin fixation; d_long_ = long diameter on transverse plane after formalin fixation; d_short_ = short diameter on transverse plane after formalin fixation; Area retraction = tissue transverse plane reduction after formalin fixation, which was calculated according to the formula (d_long_ × d_short_) / (D_long_ × D_short_)

**Supplementary Table S2** Microscopic extension (ME) and prevalence of infiltrating cells for each patient

| Case no. | Grade | ME_max_  (cm) | Mean percentage of tumor cells in the surrounding brain tissue (range, cm) | | | | | | | | | | | |
| --- | --- | --- | --- | --- | --- | --- | --- | --- | --- | --- | --- | --- | --- | --- |
|  |  |  | 0-0.25 | 0.25-0.50 | 0.50-0.75 | 0.75-1.00 | 1.00-1.25 | 1.25-1.50 | 1.50-1.75 | 1.75-2.00 | 2.00-2.25 | 2.25-2.50 | 2.50-2.75 | 2.75-3.00 |
| 1 | III | 1.34 | 46(26-57) | 24(19-29) | 14(8-21) | 5(4-8) | 3(2-5) | 2(2-4) | 0 | 0 | 0 |  |  |  |
| 2 | III | 0.98 | 54(47-61) | 25(19-34) | 14(6-21) | 6(0-10) | 0 | 0 | 0 | 0 | 0 | 0 |  |  |
| 3 | III | 1.45 | 42(41-43) | 31(29-33) | 16(12-21) | 4 (4-6) | 2(1-4) | 1(1-2) | 0 | 0 | 0 | 0 | 0 |  |
| 4 | III | 1.98 | 28(22-34) | 21(17-26) | 16(15-19) | 12(7-16) | 8(6-11) | 7(4-9) | 4(3-6) | 2(1-3) | 0 | 0 | 0 |  |
| 5 | III | 0.96 | 56(53-58) | 23(20-25) | 16(8-17) | 5(5-6) | 0 | 0 | 0 | 0 | 0 | 0 | 0 |  |
| 6 | III | 1.25 | 49(45-51) | 29(25-31) | 16(10-18) | 4(4-5) | 2(1-3) | 0 | 0 | 0 | 0 | 0 |  |  |
| 7 | III | 0.75 | 77(61-78) | 19(15-23) | 4(3-5) | 0 | 0 | 0 | 0 | 0 | 0 | 0 | 0 |  |
| 8 | III | 0.63 | 61(49-70) | 35(28-39) | 5(1-8) | 0 | 0 | 0 | 0 | 0 | 0 | 0 |  |  |
| 9 | III | 1.06 | 44(38-46) | 29(20-33) | 18(15-19) | 8(4-11) | 1(0-5) | 0 | 0 | 0 | 0 | 0 | 0 |  |
| 10 | III | 2.87 | 35(26-42) | 25(19-32) | 15(12-18) | 8(6-12) | 5(2-7) | 4(2-7) | 2(1-5) | 2(0-5) | 1(0-3) | 1(0-2) | 1(0-1) | 1(0-1) |
| 11 | III | 1.48 | 52(48-55) | 26(24-28) | 10(7-12) | 5(3-7) | 5(3-6) | 4(2-5) | 0 | 0 | 0 | 0 |  |  |
| 12 | III | 1.49 | 36(27-45) | 26(22-35) | 18(15-23) | 10(5-17) | 5(0-11) | 1(0-5) | 0 | 0 | 0 | 0 | 0 |  |
| 13 | III | 1.52 | 54(49-55) | 21(19-23) | 10(10-13) | 7(7-9) | 5(5-6) | 3(2-3) | 1(0-1) | 0 | 0 | 0 |  |  |
| 14 | III | 1.36 | 56(51-68) | 24(17-26) | 13(13-15) | 4(3-8) | 2(1-2) | 1(0-2) | 0 | 0 | 0 | 0 | 0 | 0 |
| 15 | III | 1.31 | 49(38-53) | 27(22-29) | 15(13-20) | 4(4-8) | 4(2-5) | 2(0-2) | 0 | 0 | 0 | 0 | 0 |  |
| 16 | III | 1.88 | 39(30-48) | 25(17-26) | 20(19-22) | 13(9-14) | 2(0-5) | 1(0-2) | 1(0-2) | 1(0-1) | 0 | 0 | 0 | 0 |
| 17 | III | 1.28 | 67(46-71) | 24(16-29) | 8(8-12) | 7(5-9) | 5(2-9) | 5(0-8) | 0 | 0 | 0 | 0 | 0 | 0 |
| 18 | IV | 1.50 | 35(34-37) | 22(21-24) | 17(15-19) | 12(11-15) | 8(6-9) | 4(3-4) | 0 | 0 | 0 | 0 | 0 |  |
| 19 | IV | 1.89 | 46(39-55) | 33(27-38) | 13(10-17) | 5(4-7) | 3(1-4) | 2(2-3) | 1(0-2) | 1(0-1) | 0 | 0 | 0 | 0 |
| 20 | IV | 2.60 | 51(47-55) | 15(14-16) | 12(11-13) | 8(8-9) | 6(5-6) | 4(2-5) | 2(1-3) | 1(0-2) | 1(0-1) | 1(0-1) | 1(0-1) | 0 |
| 21 | IV | 1.88 | 45(43-50) | 20(19-23) | 12(11-15) | 8(7-10) | 5(3-6) | 4(3-4) | 2(0-4) | 1(1-2) | 0 | 0 |  |  |
| 22 | IV | 2.83 | 37(27-46) | 14(11-17) | 13(10-15) | 11(8-14) | 7(4-10) | 5(3-7) | 4(1-6) | 4(1-6) | 2(0-3) | 2(0-2) | 1(0-1) | 1(0-1) |
| Case no. | Grade | ME_max_  (cm) | Mean percentage of tumor cells in the surrounding brain tissue (range, cm) | | | | | | | | | | | |
|  |  |  | 0-0.25 | 0.25-0.50 | 0.50-0.75 | 0.75-1.00 | 1.00-1.25 | 1.25-1.50 | 1.50-1.75 | 1.75-2.00 | 2.00-2.25 | 2.25-2.50 | 2.50-2.75 | 2.75-3.00 |
| 23 | IV | 1.75 | 34(33-39) | 22(24-28) | 18(15-25) | 11(9-13) | 4(3-5) | 2(1-3) | 1(0-3) | 0 | 0 | 0 | 0 |  |
| 24 | IV | 1.65 | 47(46-48) | 32(31-33) | 11(10-12) | 4(3-5) | 3(2-4) | 2(1-3) | 1(0-2) | 0 | 0 |  |  |  |
| 25 | IV | 1.74 | 55(54-58) | 22(21-25) | 7(6-8) | 7(6-8) | 5(4-6) | 3(2-4) | 1(0-1) | 0 | 0 | 0 |  |  |
| 26 | IV | 2.39 | 40(26-67) | 17(12-30) | 15(10-21) | 10(10-13) | 6(6-9) | 6(6-9) | 4(3-7) | 2(2-5) | 2(0-4) | 2(0-2) | 0 | 0 |
| 27 | IV | 2.17 | 42(30-50) | 35(31-37) | 8(8-12) | 6(4-7) | 3(3-6) | 3(1-5) | 2(0-2) | 1(0-1) | 1(0-1) | 0 | 0 |  |
| 28 | IV | 2.06 | 38(33-45) | 20(18-25) | 17(14-21) | 11(9-15) | 10(7-13) | 2(2-5) | 2(2-3) | 1(1-2) | 1(0-2) | 0 | 0 | 0 |
| 29 | IV | 2.58 | 51(42-59) | 27(18-33) | 12(8-15) | 3(3-6) | 3(3-5) | 2(1-4) | 2(2-3) | 1(1-2) | 1(0-2) | 1(0-1) | 1(0-1) |  |
| 30 | IV | 2.34 | 47(38-59) | 24(20-33) | 10(5-17) | 5(3-8) | 4(4-5) | 2(1-4) | 1(1-2) | 1(0-1) | 1(0-1) | 1(0-1) | 0 | 0 |

***Abbreviation:*** ME_max_ = the maximum of ME across different slices

**Supplementary Table S3** Baseline, immediate post-operative and long-term neurological symptoms

| Symptoms | Number of patients (%) | | | | | |
| --- | --- | --- | --- | --- | --- | --- |
|  | Total | Headache | Sensory deficits | Motor weakness | Visual deficits | Language difficulty |
| Asymptomatic | 9 (30) |  |  |  |  |  |
| Symptomatic | 21 (70) |  |  |  |  |  |
| Baseline neurological symptoms | 21 (70) | 21 (70) | 0 | 0 | 0 | 0 |
| Immediate post-operative symptoms |  |  |  |  |  |  |
| Stable | 0 | 0 | 0 | 0 | 0 | 0 |
| Improved | 3 (10) | 3 (10) | 0 | 0 | 0 | 0 |
| Worsened | 0 | 0 | 0 | 0 | 0 | 0 |
| Complete remission | 18 (90) | 18 (90) | 0 | 0 | 0 | 0 |
| New neurological symptoms | 0 | 0 | 0 | 0 | 0 | 0 |
| Long-term symptoms |  |  |  |  |  |  |
| Stable | 0 | 0 | 0 | 0 | 0 | 0 |
| Improved | 0 | 0 | 0 | 0 | 0 | 0 |
| Worsened | 0 | 0 | 0 | 0 | 0 | 0 |
| Complete remission | 3 (10) | 3 (10) | 0 | 0 | 0 | 0 |
| New neurological symptoms | 0 | 0 | 0 | 0 | 0 | 0 |
